# Preparation and Characterization of QPVA/PDDA Electrospun Nanofiber Anion Exchange Membranes for Alkaline Fuel Cells

**DOI:** 10.3390/nano12223965

**Published:** 2022-11-10

**Authors:** Asep Muhamad Samsudin, Michaela Roschger, Sigrid Wolf, Viktor Hacker

**Affiliations:** 1 Institute of Chemical Engineering and Environmental Technology, Graz University of Technology, 8020 Graz, Austria; 2 Department of Chemical Engineering, Diponegoro University, Semarang 50275, Indonesia

**Keywords:** fuel cells, AEMFCs, anion exchange membrane, poly(vinyl alcohol), PDDA, electrospinning

## Abstract

In recent years, there has been considerable interest in anion exchange membrane fuel cells (AEMFCs) as part of fuel cell technology. Anion exchange membranes (AEMs) provide a significant contribution to the development of fuel cells, particularly in terms of performance and efficiency. Polymer composite membranes composed of quaternary ammonium poly(vinyl alcohol) (QPVA) as electrospun nanofiber mats and a combination of QPVA and poly(diallyldimethylammonium chloride) (PDDA) as interfiber voids matrix filler were prepared and characterized. The influence of various QPVA/PDDA mass ratios as matrix fillers on anion exchange membranes and alkaline fuel cells was evaluated. The structural, morphological, mechanical, and thermal properties of AEMs were characterized. To evaluate the AEMs’ performances, several measurements comprise swelling properties, ion exchange capacity (IEC), hydroxide conductivity (σ), alkaline stability, and single-cell test in fuel cells. The eQP-PDD_0.5_ acquired the highest hydroxide conductivity of 43.67 ms cm^−1^ at 80 °C. The tensile strength of the membranes rose with the incorporation of the filler matrix, with TS ranging from 23.18 to 24.95 Mpa. The peak power density and current density of 24 mW cm^−2^ and 131 mA cm^−2^ were achieved with single cells comprising eQP-PDD_0.5_ membrane at 57 °C.

## 1. Introduction

Non-renewable conventional energy resources, specifically natural gas, coal, oil, and geothermal, are continuously depleting. In addition, this energy source has a negative impact on the environment, such as decreasing air quality and global warming due to greenhouse gases produced from the by-products of fossil fuel combustion [1,2]. Apart from the eminent renewable energy (e.g., biomass, wind, solar, hydropower, and tidal energy), various non-conventional renewable energy sources including fuel cells, water electrolysis, redox flow battery, salinity gradient energy (e.g., reverse electrodialysis, pressure retarded osmosis) have been continually developed to overcome these issues [3,4]. 

The fuel cell is one of the most widely studied electricity-generating technologies. Anion exchange membrane fuel cells (AEMFCs), which are one type of fuel cell, have drawn considerable interest over the past years due to several favorable characteristics among the fuel cell categories researched and developed. These include a superior oxygen reduction reaction (ORR) rate and a prospect of employing non-noble transition metals, avoiding an expensive platinum group metals (PGM) catalyst. Furthermore, low fuel crossover and reduced corrosion problems in an alkaline environment as a result of a counter direction between fuel and OH^-^ ions are also strong points of the AEMFCs [5]. 

As hydroxide-conducting polymer electrolytes, anion exchange membranes (AEMs) are one of the vital elements of AEMFCs attributable to their critical task in the performance of AEMFCs [5]. Conducting hydroxide ions, preventing electrons from passing through the internal circuit, and inhibiting fuel and oxidant crossing between electrodes are the primary roles of AEMs [6,7]. Despite its benefits, the development of AEMFCs encounters some issues. Due to the intrinsically inferior hydroxide mobility compared to a proton, AEMs have a lesser ion conductivity than proton exchange membranes (PEMs) [8]. Under extreme alkaline conditions, hydroxide-conducting charge groups in AEM tend to be unstable and rapidly degraded [9]. Some polymer dissolving processes need elevated temperature conditions and costly and toxic solvents. The membrane synthesis routes are often complex and use expensive equipment [10]. Thus, to produce high-performance AEMFCs, developing AEMs with high OH^−^ conductivity, excellent mechanical strength, and good stability are necessary [5].

To date, many researchers developed various AEMs synthesis ways to accomplish the desired performance. Among them were focusing on developing polymer materials [7,8,11,12,13], cation groups [14,15,16,17,18,19,20,21,22,23], and additives. Some researchers developed AEMs structures [24,25,26,27,28] and membrane preparation methods to enhance the AEMs’ performances [16,29,30,31]. 

Poly(vinyl alcohol) (PVA) is a white to cream-colored polymer that is characteristically odorless, tasteless, biocompatible, biodegradable, and non-toxic [32,33]. Due to its favorable properties, functionalized PVA is often used as AEMs material. Its advantages include; high water uptake thanks to its hydrophilicity, remarkable film-forming properties, low fuel crossover, and the presence of reactive functional groups, which are valuable for improving the properties of the membrane by chemical crosslinking or other chemical modification [11,34,35,36].

Among the methods of manufacturing polyelectrolyte membranes, electrospinning is becoming used widely. This method uses a high-voltage source to generate an electric field between the edge of the spinneret and the collector of the electrospinning device. At a certain level of electric field intensity, a conical-shaped Taylor cone will form at the edge of the spinneret. After the intensity exceeds the polymer drop surface tension, a solution jet will be discharged from the edge of the Taylor cone. On its way to the collector, the solution jet will evaporate and solidify to form nanofibers on the collector [37]. By electrospinning, fiber mats with high porosity and specific surface area can be produced. The average fiber diameter is frequently less than 200 nm [38,39]. Electrospinning is beneficial for providing uniaxial alignment of the polymer chains formed in nanofibers. This structure can enhance the mechanical properties of AEMs. Another advantage of electrospinning is to promote the establishment of interconnected networks, which enhances hydroxide conduction [37,40,41]. 

Park et al. prepared composite AEMs by electrospinning different polymers simultaneously, namely polyphenylsulfone and chloromethylated polysulfone. With this method, the ion exchange capacity of 2.47 mmol g^−1^ and maximum conductivity of 0.040 S cm^−1^ at 23 °C were achieved [42]. Wang et al. fabricated electrospun nanofiber AEMs using poly(aryl ether sulfone) as a backbone and guanidium as a cation side chain. The nanofiber mats were then filled with (vinylbenzyl)trimethylammonium chloride (VBTC)-based solution to acquire a composite anion exchange membrane. As the result, the conductivity of the electrospun AEMs is much higher than the casting membranes. Meanwhile, water uptake and swelling degree gave the opposite result compared to the conductivity [43]. Gong et al. utilize imidazolium-functionalized polysulfone (IMPSF) as fibers and matrix to produce an anion exchange membrane by electrospinning. The anionic conductivity of the electrospun AEMs improves remarkably by 100-fold at RH 40% and 1.7-fold at RH 100% compared to cast membranes. The membrane reveals a low swelling degree, even with high water uptake compared to cast AEMs, which is favorable for AEMs development [16].

In this work, we successfully fabricated and characterized the electrospun AEMs based on QPVA and poly(diallyldimethylammonium chloride) (PDDA). The fibers were prepared from commercial QPVA. To convert the electrospun mats into dense membranes, the combination of QPVA and PDDA acts as the matrix that fills in the voids between the fibers. PDDA, as a hydrophilic and eco-friendly polymer, possesses quaternary ammonium groups as charge carriers of hydroxide [44,45]. Additionally, the cyclic structures of poly(diallyldimethylammonium chloride can form a substantial steric hindrance, hampering the degradation of functional groups by S_N_2 nucleophilic substitution in the alkaline condition [46,47]. The effect of different mass ratios of QPVA/PDDA as a matrix on the membranes and alkaline fuel cells was investigated.

## 2. Materials and Methods

### 2.1. Materials

**Figure 1:**
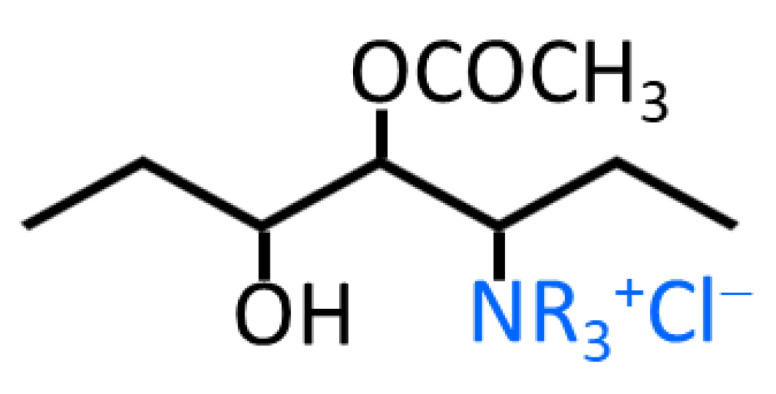
Chemical structure of Gohsenx^TM^ K-434 [48].

Quaternary ammonium poly(vinyl alcohol) (QPVA, viscosity: 18–22 mPa.s, 85.5–88.0% hydrolyzed) was obtained from Mitsubishi Chemical Corporation with a trademark of Gohsenx^TM^ K-434 (Tokyo, Japan). Figure 1 illustrates the chemical structure of Gohsenx^TM^ K-434. Poly(diallyldimethylammonium chloride) (PDDA, 20 wt.% in H_2_O, viscosity of 60–180 mPa.s, molecular weight of 400,000–500,000 g mol^−1^) and glutaraldehyde (GA, 25 wt.% in H_2_O) was purchased from Sigma-Aldrich (Steinheim, Germany). Anion exchange ionomer fumion^®^ FAA-3 solution (10 wt.%, in NPM), fumasep^®^ FAA-3-50 membrane, carbon cloth (thickness of 0.406 mm, ELAT—Hydrophilic Plain Cloth), and carbon paper (Sigracet 29 BC, thickness of 0.235 mm) were purchased from Fuel Cell Store (College Station, TX, USA). Commercial PtRu/C catalyst (HiSPEC^®^ 10000, platinum, nominally 40%, ruthenium, nominally 20% on carbon black) was obtained from Alfa Aesar (Kandel, Germany). Ultra-pure water (UPW, 18 MΩ-cm) was generated using the Barnstead E-PURE 4-Module system.

### 2.2. Preparation of Electrospun AEMs 

QPVA fiber mats were prepared by electrospinning method using electrospinning apparatus (TL-Pro, Tongli tech, Shenzhen, China). Typically, a certain quantity of QPVA GOHSENX^TM^ K-434 was dissolved in UPW with constant stirring at 80–90 °C to obtain 12 wt.% QPVA solution. The prepared solution was then filled into a 10 mL plastic syringe equipped with a spinneret needle. Subsequently, a high voltage of 20 kV was applied between the spinneret and the drum collector covered by aluminum foil substrate. The distance between the spinneret and the drum collector was placed at 10 cm. The electrospinning process is conducted overnight at a flow rate of 0.5 mL/hour at ambient temperature with a relative humidity of 50–60%. The QPVA fiber mats were heated (130 °C, 1 h) and then immersed in a crosslinking agent solution (2.5 wt.% GA and a small quantity of HCl in acetone) to promote physical and chemical crosslinking among QPVA polymer chains. 

**Figure 2:**
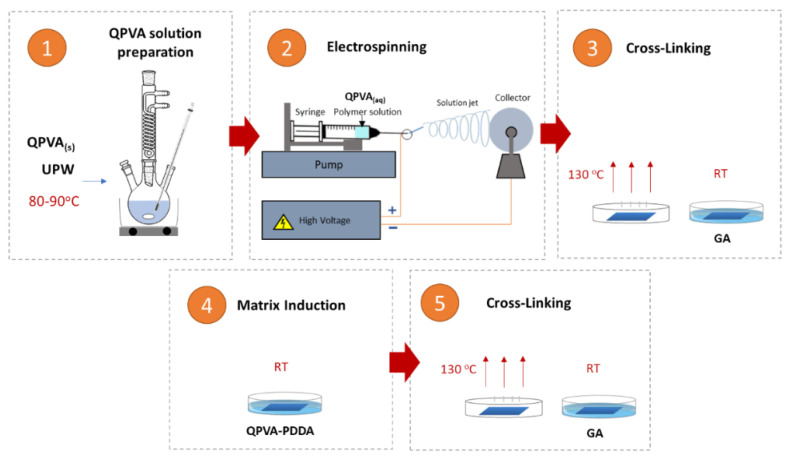
Schematic of preparation procedure.

Since dense incorporated fibers membrane is required to avoid fuel crossover, interfiber voids require to be filled. Thus, QPVA fiber mats were immersed in different QPVA/PDDA solution compositions at ambient conditions resulting in dense AEMs (eQPVA-x). Crosslinking was then performed again for dense AEMs to promote further crosslinking between QPVA chains. QPVA composite membrane samples were then named according to Table 1. Figure 2 depicts the AEMs preparation procedures.

### 2.3. Chemical Structure Characterization

The FTIR analysis was conducted using an IR-Bruker ALPHA spectrometer to assess the primary functional group of the AEMs. The IR spectra were acquired in 4 cm^−1^ resolution on the wavenumber of 500–4000 cm^−1^. The IR spectra of the resulting AEMs are presented in the form of plots of absorbance vs. wavenumbers.

### 2.4. Morphology

To identify the morphology of the AEMs, SEM (Zeiss Supra 55VP) measurement was performed. The measurement was carried out at 15 kV voltage. SEM was used to obtain the surface and cross-section of the membranes. The nanofiber diameter of the electrospun mats was measured using ImageJ software based on SEM images.

### 2.5. Thermal Stability

Since alkaline fuel cells usually operate between 70 °C and 120 °C [49], AEMs should have good stability at working temperature. In order to evaluate the thermal stability of AEMs, thermogravimetric analysis (TGA) was performed. Thermogravimetric analysis (TGA) (STA 449 C, Netsch, Selb, Germany) was used to appraise the thermal stability of membranes. The analysis was carried out at a range of 25–600 °C min^−1^ with a heating rate of 10 °C under an N_2_ flow rate of 40 mL/min. The weight loss of the membrane was evaluated against the increase in temperature.

### 2.6. Mechanical Properties

One of the desirable properties of the AEMs is that the AEMs should have exceptional mechanical properties. The membrane should be able to withstand the pressure during membrane electrode assembly (MEA) preparation and resist mechanical degradation of fuel cell operation due to physical and chemical stresses [50]. A universal testing machine (ZwickRoell Z010) was used to determine the tensile strength (TS) and elongation at break (Eb) of the membranes with a 5 mm min^−1^ strain rate at room temperature. In addition to comparing the mechanical properties of the AEMs under varying matrix filler concentrations, the mechanical properties of the membrane under dry and wet conditions were also compared.

### 2.7. Swelling Properties

Water uptake (WU) and swelling degree (SD) were accomplished to evaluate the swelling properties of the AEMs. The water uptake was defined by calculating the weight change in the AEMs caused by water immersion. The swelling degree was assessed after comparing the AEMs volume caused by water immersion as well. Before water immersion, the AEMs’ weight (W_d_) and volumes (V_d_) were measured. Subsequently, the AEMs were immersed in UPW for 24 h under ambient conditions. The weight (W_w_) and the volume (V_w_) of the AEMs after the immersion process were quantified instantly after carefully eradicating the remaining water on the surface of the membranes. Equations (1) and (2) were used to calculate the WU and SD of AEMs, respectively.

(1) $$\mathrm{WU}=\frac{W_{w}\;-\;W_{d}}{W_{d}}\;\times \;100\%$$

(2) $$\mathrm{SD}=\frac{V_{w}\;-\;V_{d}}{V_{d}}\;\times \;100\%$$

### 2.8. Ion Exchange Capacity 

The ion exchange capacity (IEC) was evaluated by a back-titration. Initially, the weight of AEMs samples (W_d_) was measured. Subsequently, the AEMs were immersed in 1.0 M KOH solution for 24 h to perform an alkalization to convert the membranes from Cl^−^ into OH^−^. The remaining KOH residue of membranes was then removed by soaked the AEMs in UPW for 24 h. After that, the AEMs were immersed in 0.1 M HCl solution (C_HCl_) for a further 24 h. The titration was conducted automatically using a titrator (Titroline^®^ 7800, SI Analytics, Xylem Analytics, Mainz, Germany) using 0.1 M NaOH solution as a titrant. After reaching the equivalence point, the consumed volumes of NaOH without membrane as the blank (V_b_) and with AEMs (V_m_) were documented. Equation (3) was used to calculate the IEC of AEMs.

(3) $$\mathrm{IEC}=\frac{\left(V_{b}\;-\;V_{m}\right)\cdot C_{\mathrm{HCl}}}{w_{d}}$$

### 2.9. Hydroxide Conductivity 

The hydroxide conductivity of the AEMs was assessed by electrochemical impedance spectroscopy (EIS) measurement. Initially, the membranes were immersed in a 1.0 M KOH solution for 24 h. Subsequently, the membrane was soaked in UPW to eradicate excess KOH. The membrane samples were then positioned in the Bekktech BT110 LLC four-electrode conductivity clamp (Scribner Associates, Southern Pines, NC, USA) which was further immersed in UPW during measurement. The Gamry Reference 600 potentiostat was used to determine the impedance of membranes with a frequency range of 0.1 Hz–10 kHz and an amplitude of 50 mV. The AEMs resistance (R_m_) was acquired from the intercept of the Nyquist curve with the real axis Z_real_. Equation (4) was employed to determine the OH^−^ conductivity.

(4) $$\sigma =\frac{d}{R_{m}\cdot T\cdot W}$$

where d is the distance between electrodes sense, T is the thickness of AEMs in a wet state, and W is the width of the AEMs. 

### 2.10. Alkaline Stability Test

The alkaline stability of the AEMs was assessed by submerging the membrane in 6 M KOH at 80 °C for a period of time. At specific time intervals, the variation in OH^−^ conductivity was recorded. After washing the AEMs sample with ultra-pure water to remove a free KOH on the surface of the AEMs, the OH^−^ conductivity of the AEMs was measured at 30 °C.

### 2.11. Single-Cell Performance Test

The AEMs’ performance in fuel cells was examined using an alkaline direct ethanol cell (ADEFC). At first, the AEMs were immersed in 1.0 M KOH for 24 h and rinsed with ultra-pure water before performing the single-cell test. An automatic ultrasonic spray coater (Sonotek, Milton, NY, USA) was used to prepare electrodes through anode and cathode catalyst ink slurries deposition onto the gas diffusion layer (GDL). The anode was prepared by spraying the PdNiBi/C catalyst ink slurry (synthesized by the modified instant reduction method [51]) on carbon cloth resulting in 0.75 mg cm^−2^ metal loading. On the other hand, the cathode was prepared by spraying a commercial PtRu/C catalyst ink slurry onto carbon paper resulting in 0.5 mg cm^−2^ metal loading. Both inks consisted of a catalyst, an isopropanol/water (7/3) solution, and an anion-exchange ionomer. 

The membrane electrode assembly (MEA) was assembled by placing the AEM between the electrodes and the whole MEA inside a self-made ADEFC. A mixture of 3.0 M ethanol and 5.0 M KOH with a flow rate of 5 mL min^−1^ was employed as anode fuel, whereas oxygen (purity of grade 4.5 [99.995%], 25 mL min^−1^) was applied as a cathode feed gas. The measurements were performed for the best self-made AEMs and the commercial FAA-3-50 AEMs, as a reference, at room temperature and elevated temperatures (36 °C, 44 °C, 53 °C, and 57 °C). A Reference 600TM potentiostat (Gamry Instruments) was used to determine the cell potentials, current densities, and power density.

## 3. Results

### 3.1. AEMs Preparation

**Figure 3:**
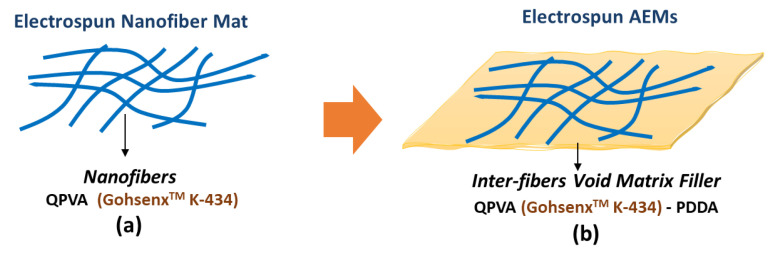
Schematic of (**a**) electrospun nanofiber mat, (**b**) electrospun AEMs.

In this work, electrospun QPVA anion exchange membranes were fabricated with two main steps (Figure 3). Firstly, the electrospinning of QPVA solution to produce QPVA nanofiber mats. Secondly, the preparation of QPVA anion exchange membranes by incorporating a different ratio of QPVA-PDDA matrix filler into the inter-fiber voids of the QPVA nanofiber mat. 

The electrospinning process can provide several benefits. These benefits include providing uniaxial alignment of the polymer chains, which can improve the mechanical properties of AEMs. Another advantage of electrospinning is it promotes the establishment of interconnected networks, which can raise hydroxide conduction [37,40,41]. In this work, the electrospinning process was performed according to the process parameters in Table 2. When the QPVA solution concentration and voltage were too low, or the flow rate of the solution jet was too high, droplets were generated during the electrospinning procedure. Infrequently, these droplets dropped from the needle tip or were transported through the liquid jet and adhered to the collector, causing defects in the nanofiber mat. If the voltage was too high, this caused the liquid jet to become unstable, leading to a poor nanofiber mat result. When the relative humidity is too low, the polymer solution at the tip of the spinneret dries easily and becomes partially or completely clogged, preventing the formation of the desired Taylor cone. In contrast, excessive humidity makes it more difficult for the nanofiber mat to dry. The appearance of nanofiber mat with poor and good results can be seen in Figure S1. 

Since a dense incorporated fiber membrane is required to avoid fuel crossover, interfiber voids must be filled. Thus, electrospun QPVA nanofiber mats were immersed in different compositions of QPVA/PDDA matrix solution at ambient conditions resulting in denser AEMs (eQPVA-x). Crosslinking was then performed again for AEMs to promote further crosslinking between QPVA chains.

### 3.2. Chemical Structure

**Figure 4:**
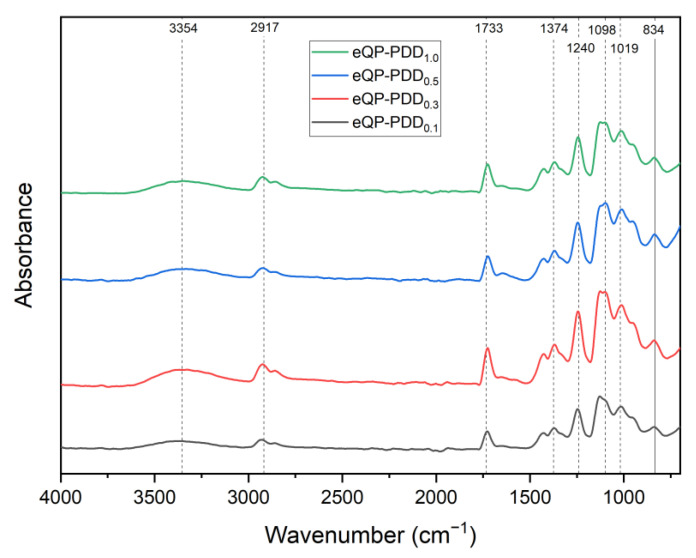
IR spectra of eQP-PDDx AEMs.

In order to identify the primary functional groups that are present in AEMs, FTIR analysis was performed. Figure 4 depicts the typical IR spectra of eQP-PDD_x_ anion exchange membranes. The spectra exhibit peaks at 3354 and 1019 cm^−1^, which can be identified as the stretching vibration of −OH groups and C–H from the QPVA. The peak around 2917 cm^−1^ appears from the C–H in-plane bend. The intensity around 1773 cm^−1^ presents as a C=O stretch, which is likely part of ester groups that arises due to partial hydrolysis in the manufacture of PVA [32,35]. The peak at the wavenumber around 1374 cm^−1^ indicates the stretching vibration of the C–O group. An intensity around 1240 cm^−1^ refers to the C–O–C stretch group indicating the formation of covalent bonds between the CHO groups of GA and the H groups of QPVA. The peaks that appear at 1098 cm^−1^ and 834 cm^−1^ fit in the C–N stretch groups from QPVA or PDDA and the N-H bend from cation groups of QPVA. 

### 3.3. Morphology

**Figure 5:**
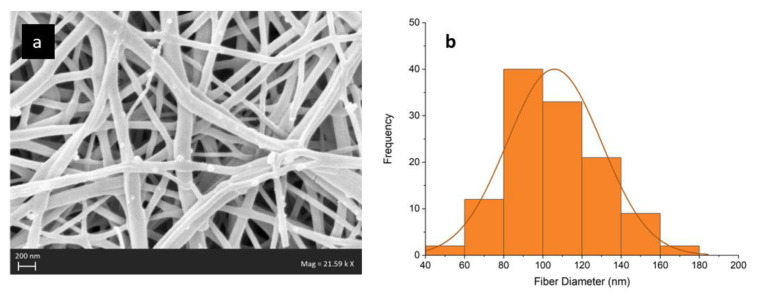
(**a**) SEM Image and (**b**) size distribution of electrospun nanofiber mat of QPVA (without matrix).

The surface morphology of the electrospun QPVA AEMs can be observed in Figure 5a. The solid and uniform polymer fibers with random orientation and no significant beads are formed. Figure 5b depicts the size distribution of the membrane. The fibers have a size distribution in the range of 50–179 nm with an average fiber diameter of 101.98 nm, which is considered nanofibers [52]. Inter-fiber void space (porous) of the membranes is visible. To be applied in fuel cells, this inter-fiber void space of the membrane must be filled with a filler matrix to become a dense membrane. This filler matrix serves to prevent the passage of gas through the membrane (fuel or oxidant), known as gas crossover. Fuel crossover or fuel permeability through anion exchange membranes is undesired for fuel cells. This process can trigger voltage loss owing to mixed potential resulting from the oxidation of permeated fuel. In addition, fuel crossover can generate additional heat, and peroxide leads to a degradation of the fuel cell [53,54,55].

**Figure 6:**
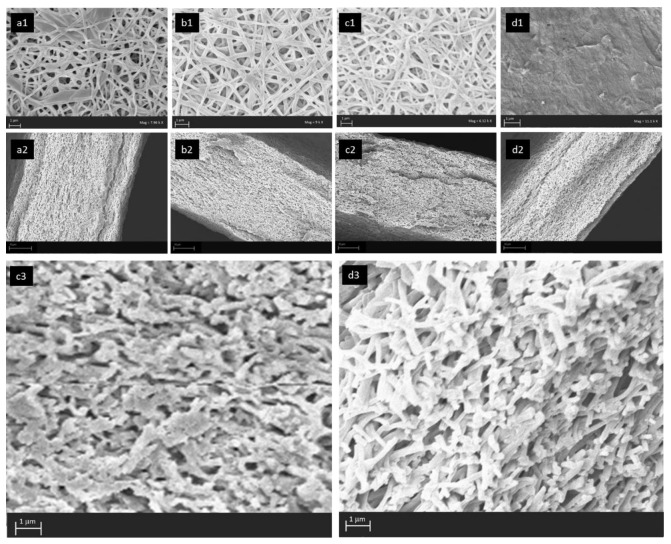
SEM images of surface morphology (no. 1) and cross-section (no. 2 and 3) of (**a**) eQP-PDD_0.1_, (**b**) eQP-PDD_0.3_, (**c**) eQP-PDD_0.5_, and (**d**) eQP-PDD_1.0_.

Figure 6 depicts the surface and cross-section morphology of eQP-PDD_0.1_, eQP-PDD_0.3_, eQP-PDD_0.5_, and eQP-PDD_1.0_ anion exchange membranes. The QPVA nanofiber membranes are filled with a filler matrix composed of QPVA and PDDA with different mass ratios. Since PDDA does not possess functional groups that can be crosslinked with GA solution, QPVA is mixed with PDDA. With this approach, PDDA can be trapped in the interchain pores by the crosslinked QPVA, which can then be further crosslinked with the unreacted active functional group of the QPVA nanofiber [56]. According to surface morphology (Figure 6a–d), the porosity of membranes decreases with the rise of PDDA concentration in the AEMs matrix. The inter-fibers voids on the electrospun mats were filled with the matrix, causing the inter-fibers voids to become more faintly visible with the addition of PDDA concentration. Eventually, the voids between fibers are no longer visible on the surface of eQP-PDD_1.0_ AEMs. This is because the concentration of PDDA (10 wt.%) is two-fold compared to the concentration of QPVA (5 wt.%) in the matrix. Moreover, the viscosity of PDDA is much larger than that of QPVA. SEM images for membrane cross-sections (Figure 6a–d2) also generally show a phenomenon similar to surface images (Figure 6d–d). The membrane porosity declines when the ratio of PDDA to QPVA rises from 0.1 to 0.5 and then increases again at a ratio of 1.0. (i.e., more porous). The comparison of the cross-sectional images between eQP-PDD_0.5_ and eQP-PDD_1.0_ at higher magnification can be seen in Figure 6c2,d2. This phenomenon is probably because the viscosity of the matrix increases with the rise of the PDDA/QPVA mass ratio, leading it to be more difficult for the matrix to penetrate the interfiber voids of the nanofibers mat.

### 3.4. Thermal Stability

**Figure 7:**
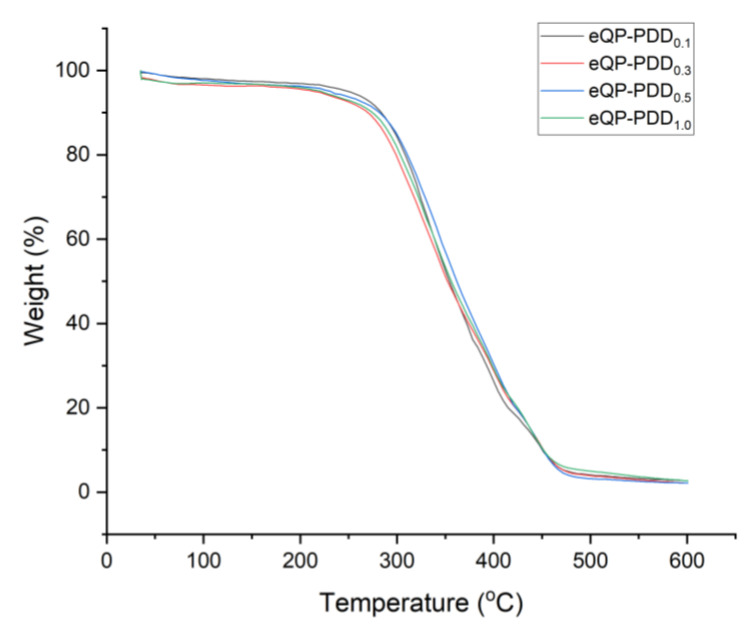
Thermogravigrams of eQP-PDD_x_ AEMs.

Figure 7 illustrates the thermal characteristics of eQP-PDD_0.1_, eQP-PDD_0.3_, eQP-PDD_0.5_, and eQP-PDD_1.0_ anion exchange membranes. According to the figures, all thermogravigrams exhibit a similar tendency with three significant weight loss phases. The first phase, which appears in the range of 30–105 °C and has a weight loss of around 4%, denotes the release of water molecules from AEMs as well as the absorption of moisture from the surrounding environment. In the second phase, temperatures range from 205 °C to 305 °C. At this stage in the TGA, the overall mass loss was around 12%. This may be caused by various factors, including the breakdown of some C–C and C–O bonds from PDDA and QPVA, as well as the break of crosslinking bonds and quaternary ammonium cationic group degradation [57,58,59]. The last phase, which develops between 315 °C and 475 °C with a total weight loss of around 68%, is associated with the polymer backbones degradation of QPVA and PDDA [46]. The thermogravimetric analysis results indicate that eQP-PDD_x_ AEMs have adequate thermal stability to be employed in low-temperature fuel cells.

### 3.5. Mechanical Properties

The mechanical attributes (e.g., tensile strength and elongation at break) of the AEMs as a polymer electrolyte are crucial aspects that must be considered while constructing the MEA of the fuel cells. The membrane must retain the necessary mechanical strength to accommodate the pressing during fuel cell assembly and operation. Based on Table 3, the higher the fraction of PDDA to QPVA in the matrix, the higher the membrane thickness until the membrane eQP-PDD_0.5_. However, there is a decrease in thickness in eQP-PDD_1.0_ due to the high viscosity of the matrix. Thus, it is difficult for the matrix to penetrate the inter-fiber voids of the nanofibers mat. 

**Figure 8:**
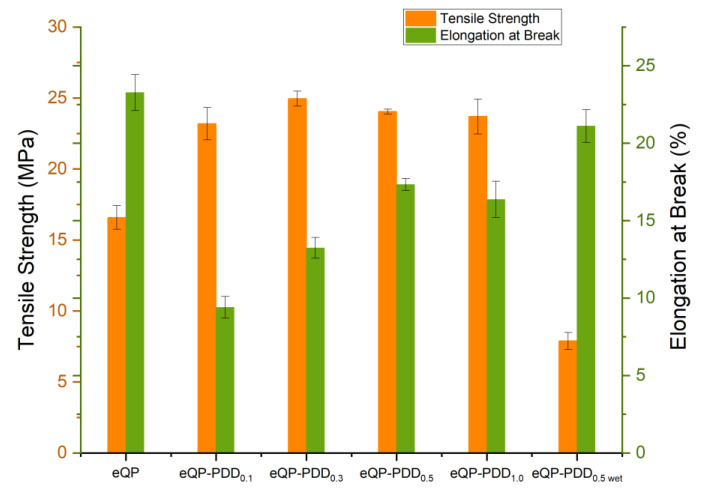
Tensile strength and elongation at break of eQP-PDD_x_ AEMs.

The tensile strength (TS) and elongation at break (Eb) of AEMs with various QPVA/PDDA mass ratios have been investigated in regard to their mechanical properties (Figure 8 and Table 3). The eQP AEMs possess the lowest tensile strength and the highest elongation at break compared to other membranes in dry conditions due to the high porosity of this membrane [60]. Along with adding a PDDA/QPVA matrix ratio of 0.1, the TS of the eQP-PDD_0.1_ AEMs increased by about 40% but decreased the Eb compared to without matrix filler. This implies that the introduction of inter-fiber matrix filler can strengthen the membrane and make it more rigid. When the PDDA/QPVA matrix ratio was increased further, the TS of the dense eQP-PDDx AEMs raised between 23.18 and 24.95 Mpa and raised the Eb as well. 

Table 3 and Figure 8 also provide a comparison of the AEMs in dry (eQP-PDD_0.5_) and wet conditions (eQP-PDD_0.5 wet_). The tensile strength of eQP-PDD_0.5_ AEMs declined while the Eb increased in the hydrated state. The presence of water in the AEMs has a plasticizing impact, which causes the AEMs to become pliable and weakens their strength [50].

### 3.6. Swelling Properties

Achieving a high OH conductivity in AEMs requires the presence of water. Water clusters can serve as anion transport routes within the anion-exchange membrane, hence enhancing the ionic conductivity [34]. In contrast, excessive water uptake might cause severe membrane swelling, resulting in reduced dimensional stability, diminishing contact between AEMs and the electrodes’ active layer, and thus limiting the fabrication of membrane electrode assemblies (MEA) [61]. It is possible that the massive swelling may dilute the number of charge carriers to the point where the membrane loses some of its ability to conduct ions [62]. To evaluate the behavior of AEMs after absorbing some water, water uptake (WU) and swelling degree (SD) were calculated. The WU and SD were calculated by measuring the change in weight and dimension due to the water absorption. 

**Figure 9:**
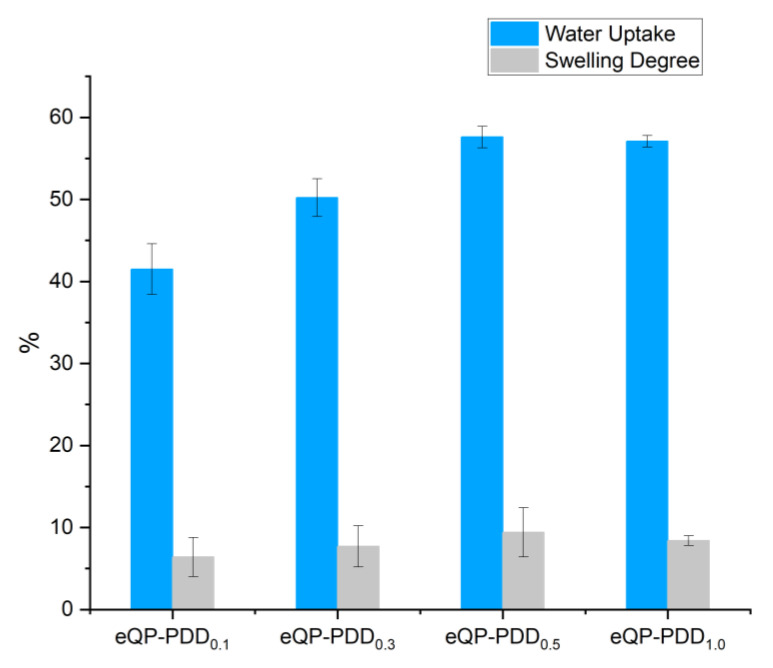
WU and SD of eQP-PDD_x_ AEMs.

The water uptake and swelling degree of the eQP-PDDx AEMs are depicted in Table 3 and Figure 9. The WU of eQP-PDD_0.1_ AEMs is 41.5%, which enhances as the PDDA/QPVA matrix filler ratio rises to 0.3 and 0.5, leading to a WU of 50.2% and 57.4%, respectively. After the PDDA/QPVA matrix filler ratio was increased two-fold to 1.0 (eQP-PDD_1.0_), the membrane water uptake decreased slightly. The addition and reduction of water uptake are influenced by the quantity of conducting cation ions contained in the AEMs, specifically from the quaternary ammonium groups in QPVA and PDDA [56]. 

The results of the swelling degree measurement are also in accordance with the water uptake findings with a similar data trend. However, the value of the swelling degree is much smaller than the water uptake, which ranges from 6.4 to 9.4. This means that the nanofiber structure can suppress dimensional changes while still absorbing adequate water. 

### 3.7. Ion Exchange Capacity and Hydroxide Conductivity

**Figure 10:**
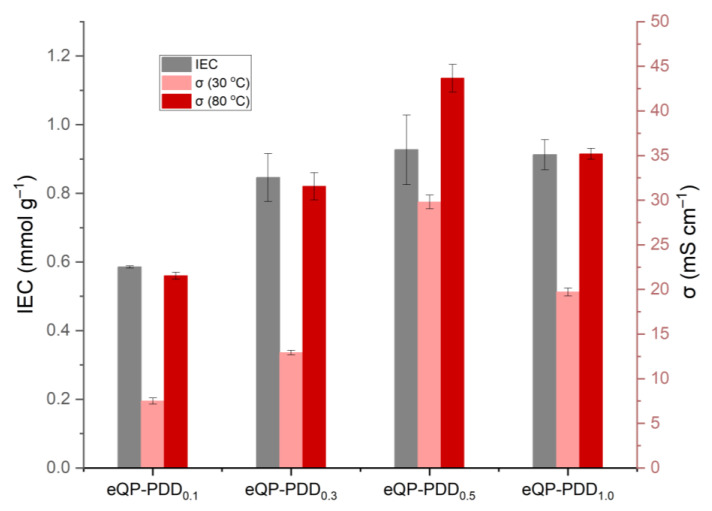
Ion exchange capacity and hydroxide conductivity of eQP-PDD_x_ AEMs.

The ion exchange capacity (IEC), which is a crucial attribute of the AEMs, provides detail about the quantity of ion-exchangeable groups present in the membranes, which is significant for conducting hydroxide [46]. A high ion exchange capacity generally implies a high OH^−^ conductivity. Table 3 and Figure 10 depict the IEC and hydroxide conductivity (σ) of eQP-PDDx AEMs at 30 °C and 80 °C room temperature, respectively. The IEC of eQP-PDD_0.1_ AEMs is 0.59 mmol g^−1^. The IEC rises by about 44% and 58% with the introduction of the PDDA/QPVA matrix filler ratio of 0.3 and 0.5, respectively. The highest IEC value is obtained at a PDDA/QPVA matrix ratio of 0.5 (eQP-PDD_0.5_), i.e., 0.93 mmol g^−1^. Subsequently, IEC slightly decreased by 2% when the matrix filler ratio was increased to 1.0. The reduction in IEC of eQP-PDD_1.0_ is most likely due to the high viscosity of the matrix, which results in difficulty for the matrix to penetrate the inter-fiber voids of the nanofibers mat. As a result, the quantity of ion-exchangeable cations in the AEMs reduces, and the IEC decreases slightly.

Hydroxide conductivity is one of the principal properties of AEMs, due to the primary function of AEMs as hydroxide conductors, which corresponds to the performance of fuel cells. Table 3 shows that the hydroxide conductivities of all eQP-PDD_x_ AEMs range from 7.5 to 29.79 mS cm^−1^ at low temperatures (30 °C) and 21.53 to 43.68 mS cm^−1^ at higher temperatures (80 °C). The hydroxide conductivity was observed to rise in Table 3 and Figure 10, corresponding to the increasing IEC of membranes. The highest anion conductivity of the eQP-PDD_x_ AEMs was achieved with a PDDA/QPVA matrix filler ratio of 0.5 (i.e., 29.79 mS cm^−1^ and 43.68 mS cm^−1^ at 30 °C at 80 °C, respectively), which has the highest IEC as well. The higher the IEC, the higher the amount of quaternary ammonium as an ion-conducting cation, resulting in transporting more hydroxide and increasing ionic conductivity. Temperature increases can improve the OH^−^ mobility in AEMs. In addition, a temperature rise can drive the enlargement of free space in the AEMs, which is favorable for conducting ions [63]. Table 4 summarizes the IEC and OH^−^ conductivity reported for PVA-based AEMs at 60–80 °C.

### 3.8. Alkaline Stability

The alkaline stability has been recognized as the most significant factor inhibiting the operation of fuel cells [68]. The persistent chemical degradation of the anion-conducting polymer caused by the alkaline condition of the fuel cells’ operation is the fundamental issue of the poor stability of AEMFCs. The molecular structure of hydroxide conducting polymers that are present in AEMs and ionomers deteriorates in the severe pH conditions of AEMFCs due to the significant reactivity of OH^−^ with quaternary ammonium functional groups. This degradation leads to a detrimental reduction in the IEC of AEMs, which in turn results in a drop in conductivity, a rise in cell resistance, and a sharp decline in performance [69].

**Figure 11:**
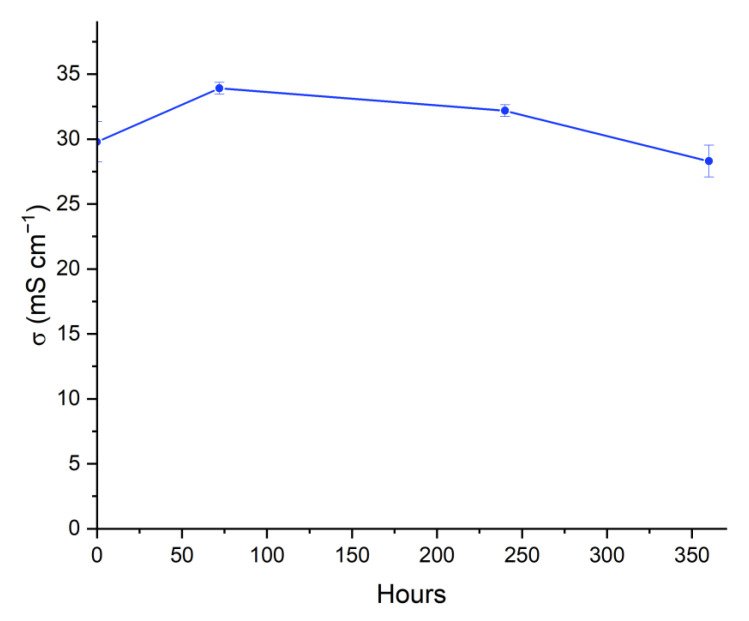
The alkaline stability of eQP-PDD_0.5_ AEMs.

The alkaline stability of the eQP-PDD_0.5_ was investigated in this work by immersing the membranes in severe conditions (i.e., 6.0 M KOH solutions at 80 °C) for a set period. Subsequently, the AEMs OH^−^ conductivity was determined at 30 °C after repeated rinses using ultrapure water to thoroughly eradicate the KOH residue on the membrane surface. Figure 11 demonstrates that the hydroxide ion conductivity of the eQP-PDD_0.5_ AEMs increases after the second measurement (64 h), which may be due to the high concentration of KOH, which penetrates the AEMs and induce a rise in OH^−^ conducting parts [44]. This may possibly be because at the initial conductivity measurement, not all Cl^−^ as the counter ion of AEMs was substituted by OH^−^ as a result of activation with 1 M KOH. Due to the higher concentration of 6 M KOH than 1 M KOH, some of the residual Cl^−^ was substituted by OH^−^ after 64 h of immersion, resulting in an increase in conductivity. 

After 240 h immersion, the membrane conductivity decreased by 5% from the previous conductivity but was still 8% higher than the initial conductivity. It is well known that AEMs can undergo chemical instability in alkaline environments, with chemical degradation predominantly caused by nucleophilic attack of OH^−^ ions on the cationic fixed charged sites. High-temperature conditions may accelerate the degradation process [70]. Reductions in OH^−^ conductivity are caused by the E_2_ Hoffman degradation and S_N_2 nucleophilic substitution process, which result in the degradation of certain functional quaternary ammonium groups [71,72]. The conductivity was reduced by 12% from before or 5% from the initial conductivity after up to 360 h of immersion, indicating that the eQP-PDD_0.5_ AEMs have good chemical stability. 

### 3.9. Single-Cell Performance

The eQP-PDD_0.5_ AEMs, which provide the maximum OH^−^ conductivity, were used to prepare an MEA. The membrane performance was evaluated in alkaline direct ethanol single test cells. The influence of temperature on AEMs performance was evaluated. A different MEA that employed a commercial FAA-3-50 AEM was fabricated to compare the membrane performance. 

**Figure 12:**
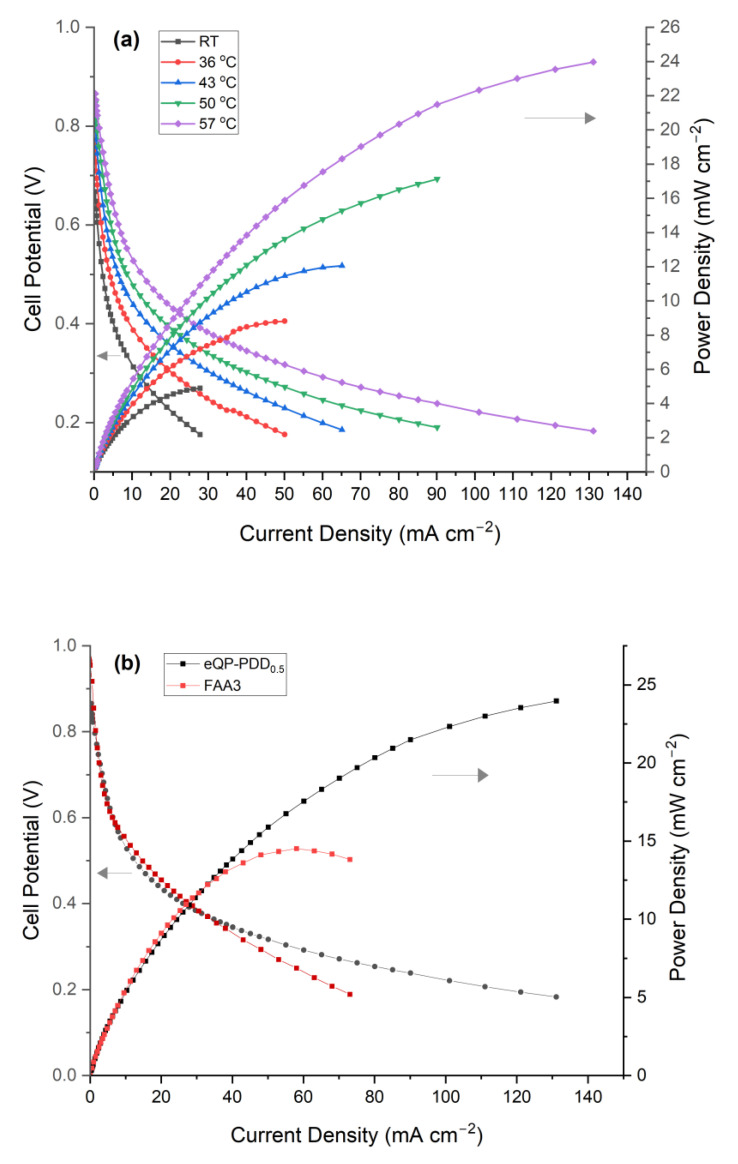
Power density and polarization curves of eQP-PDD_0.5_ AEMs. (**a**) Different temperature, (**b**) comparison with FAA-3-50 at 57 °C.

Typical curves of I-V and I-P of the eQP-PDD_0.5_ AEMs in the ADEFC are depicted in Figure 12a. As the temperatures rose, the cell voltage and maximum power density of all membranes continued to increase. The enhanced reaction kinetics at the electrodes and the rise of conductivity of the membranes inside the cell at higher temperatures are mainly accountable for these results, which correspond to similar works [56,73]. The maximum power density and current density of 24 mW cm^−2^ and 131 mA cm^−2^ were achieved with the single cell measurement comprising eQP-PDD_0.5_ membrane at 57 °C. 

A comparison of the eQP-PDD_0.5_ AEMs and commercial FAA3 AEMs performances in the alkaline cell at 57 °C is shown in Figure 12b. The MEA inside the single cell equipped with the commercial FAA-3-50 AEM generates a maximum power density and current density of 13.8 mW cm^−2^ and 73 mA cm^−2^, respectively. If we compare the performance between the eQP-PDD_0.5_ AEM and FAA-3-50 in a single cell under the same measurement conditions, we can see that the maximum power density and current density of the eQP-PDD_0.5_ AEMs are higher than that of FAA-3-50 membrane measurement.

## 4. Conclusions

A series of composite anion exchange membranes (AEMs) comprising quaternary ammonium poly(vinyl alcohol) (PVA) electrospun nanofiber and different ratios of PDDA/QPVA matrix filler have been successfully fabricated by solution casting. IR spectra successfully identified the primary functional groups of QPVA and PDDA. The three primary weight loss phases revealed by thermogravimetric analysis imply that AEMs have sufficient thermal stability to be employed in a low temperatures fuel cell. SEM analysis exhibited the nanofibers structures of eQP AEMs with an average fiber size of 101.98 nm. The membranes gradually became denser after introducing a PDDA/QPVA filler matrix. The tensile strength of the membranes rose with the incorporation of the filler matrix, with TS ranging from 23.18 to 24.95 MPa, while the elongation at break dropped and gradually increased again when the filler matrix was applied. By introducing PDDA/QPVA matrix filler of 0.5, the eQP-PDD_x_ AEMs acquired the highest water uptake, IEC and OH^-^ conductivity of 57.6%, 0.93 mmol g^−1,^ and 43.67 ms cm^−1^, respectively. After being subjected to extreme conditions (6 M KOH and 80 °C) for 360 h, eQP-PDD0.5 exhibits good alkaline stability with a 5% drop in conductivity. The maximum peak power density and current density of 24 mW cm^−2^ and 131 mA cm^−2^ were achieved with single cells comprising eQP-PDD_0.5_ membrane at 57 °C. In comparison to single cells comprising commercial FAA-3-50 membranes under identical conditions, these results are about two times higher. According to this study, eQP-PDDx AEMs are a viable alternative for application in AEMFCs.

## Figures and Tables

**Table 1:** Composition of the samples.

Sample Names *	Matrix Composition (PDD_x_) (5 wt.% QPVA: 10 wt.% PDDA)
eQP	-
eQP-PDD_0.1_	1:0.1
eQP-PDD_0.3_	1:0.3
eQP-PDD_0.5_	1:0.5
eQP-PDD_1.0_	1:1

* 12 wt.% QPVA solution was used to prepare electrospun mats for all AEMs samples.

**Table 2:** Parameter process of electrospinning.

Parameter	Value
Material	
- Concentration of QPVA	12 wt.%
Operation	
- Voltage	20 kV
- Spinneret-to-collector distance	10 cm
- Flow rate	0.5 mL/hour
Environment	
- Temperature	RT
- Relative Humidity	50–60%

**Table 3:** Physicochemical parameters of eQP-PDD_x_ AEMs.

AEMs	Thickness (μm)	IEC (mmol·g^−1^)	WU (%)	SD (%)	TS (MPa)	*E_b_* (%)	*σ* (30 °C) (mS·cm^−1^)	*σ* (80 °C) (mS·cm^−1^)
eQP	31.23	-	-	-	16.58	23.27		-
eQP-PDD_0.1_	34.33	0.59	41.5	6.4	23.18	9.40	7.50	21.53
eQP-PDD_0.3_	37.90	0.85	50.2	7.7	24.95	13.23	12.93	31.56
eQP-PDD_0.5_	45.33	0.93	57.6	9.4	24.04	17.33	29.79	43.68
eQP-PDD_1.0_	38.73	0.91	57.1	8.4	23.69	16.37	19.73	35.20
eQP-PDD_0.5 wet_	40.00	-	-	-	7.89	21.10		-

**Table 4:** Reported IEC and OH^−^ conductivity for PVA-based AEMs at 60–80 °C.

Materials	Preparation Method	IEC (mmol·g^−1^)	σ (mS·cm^−1^)	References
PVA/PDDA	Solution casting	0.89	37 (80 °C)	[34]
QPVA/PDDA	Solution casting	1.02	36.5 (60 °C)	[59]
PVA/PUB	Solution casting	1.20	9 (80 °C)	[30]
QPVA/KOH	Solution casting	1.20	30.7 (70 °C)	[64]
QPVA	Electrospinning	0.73	42 (60 °C)	[65]
PVA-PY-DLx	Solution casting	N/A	10.5 (70 °C)	[66]
PVA-HH	Solution casting	N/A	6.16 (70 °C)	[67]
eQP-PDDA_0.5_	Electrospinning	0.93	43.67 (80 °C)	This work

## Data Availability

The data that support the findings of this study are available within the article. The supplementary materials are available at the Nanomaterials website.

## Supplementary Materials

The following supporting information can be downloaded at: https://www.mdpi.com/article/10.3390/nano12223965/s1, Figure S1: Electrospun QPVA with poor result (A) and good result (B).
