# Supplementary material for: Preparation and Characterization of QPVA/PDDA Electrospun Nanofiber Anion Exchange Membranes for Alkaline Fuel Cells
Source: Nanomaterials (Basel). 2022 Nov 10;12(22):3965. doi: 10.3390/nano12223965 (PMC9693389; doi:10.3390/nano12223965)
Supplement: Supplementary file 1 [file nanomaterials-12-03965-s001.zip › nanomaterials-1983702-supplementary.pdf]

Supplementary Materials

Preparation and Characterization of QPVA/PDDA Electrospun Nanofiber Anion Exchange Membranes for Alkaline Fuel Cells

Asep Muhamad Samsudin [1,2,*], Michaela Roschger [1], Sigrid Wolf [1] and Viktor Hacker [1]

[1] Institute of Chemical Engineering and Environmental Technology, Graz University of Technology, 8020 Graz, Austria
[2] Department of Chemical Engineering, Diponegoro University, Semarang 50275, Indonesia
* Correspondence: asep.samsudin@tugraz.at

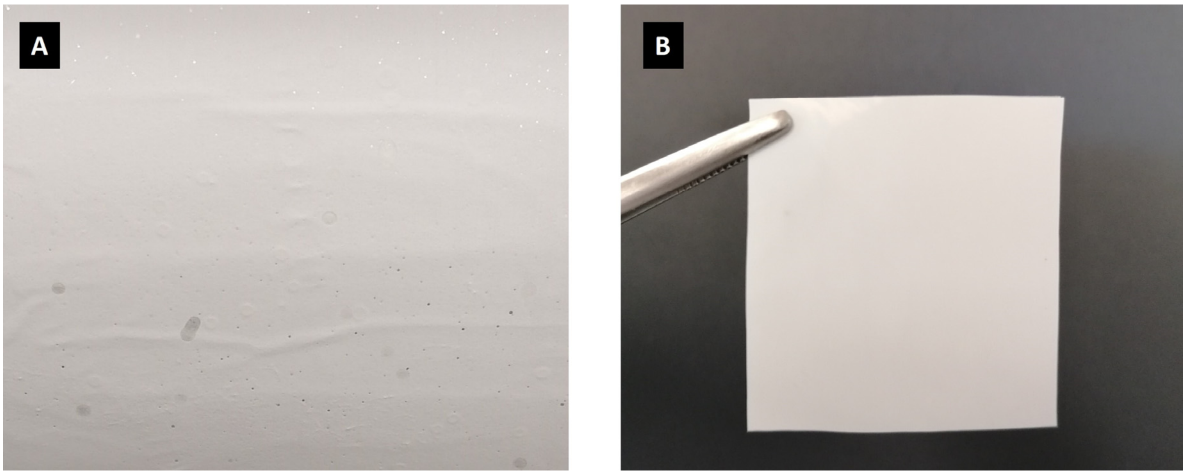

Figure S1. Electrospun QPVA with poor result (**A**) and good result (**B**).
